# A Novel Human Recombinant Lactoferrin Inhibits Lung Adenocarcinoma Cell Growth and Migration with No Cytotoxic Effect on Normal Human Epithelial Cells

**DOI:** 10.1007/s00005-021-00637-2

**Published:** 2021-11-08

**Authors:** Paulina Olszewska, Barbara Pazdrak, Marian L. Kruzel

**Affiliations:** 1 Department of Pharmaceutical Chemistry, Drug Analysis and Radiopharmacy, Faculty of Pharmacy, Medical University of Lodz, Lodz, Poland; 2 The University of Texas MD Anderson Cancer Center, Houston, TX USA; 3 PharmaReview Corporation, Houston, TX USA

**Keywords:** Recombinant human lactoferrin, Lung cancer, Glycosylation, Apoptosis

## Abstract

Lung cancer remains the leading cause of cancer death worldwide. Despite the recent advances in cancer treatment, only a subset of patients responds to targeted and immune therapies, and many patients developing resistance after an initial response. Lactoferrin (Lf) is a natural glycoprotein with immunomodulatory and anticancer activities. We produced a novel recombinant human Lf (rhLf) that exhibits glycosylation profile compatible with the natural hLf for potential parenteral therapeutic applications. The aim of this study was to evaluate the anticancer effects of this novel rhLf in human lung adenocarcinoma cells and its mechanisms of action. The results showed a concentration-dependent inhibition of A549 cancer cell growth in response to rhLf. Treatment with 1 mg/ml of rhLf for 24 h and 72 h resulted in a significant inhibition of cancer cell growth by 32% and 25%, respectively. Moreover, rhLf increased fourfold the percentage of early and late apoptotic cells compared to the control. This effect was accompanied by increased levels of caspase-3 activity and cell cycle arrest at the S phase in rhLf-treated cancer cells. Furthermore, rhLf significantly attenuated A549 cell migration. Importantly, treatment of normal human bronchial epithelial (NHBE) cells with rhLf showed the cell viability and morphology comparable to the control. In contrast, chemotherapeutic etoposide induced cytotoxicity in NHBE cells and reduced the cell viability by 40%. These results demonstrate the selective anticancer effects of rhLf against lung adenocarcinoma cells without cytotoxicity on normal human cells. This study highlights a potential for clinical utility of this novel rhLf in patients with lung cancer.

## Introduction

Cancer is a growing public health issue and is still one of the greatest medical challenges worldwide (Bray et al. 2018). Lung cancer is the most frequently diagnosed malignant tumor and it is also the leading cause of cancer death among both men and women, with an estimated 1.8 million deaths occurred in 2020 (Ferlay 2020; Sung et al. 2021). Despite the recent major advances in cancer treatment by applying targeted and immune therapies, clinical studies have shown that only a subset of patients respond to checkpoint blockade treatment and targeted therapy has benefit for selected patients with specific molecular subtypes of lung cancer (Qiao et al. 2018; Yoneda et al. 2018). While the cytotoxicity of current chemotherapeutics is still a major clinical issue, a standard chemotherapy remains the most common treatment for lung cancer patients (Carbone et al. 2015). Thus, there is an urgent need to develop alternative therapeutics that would discriminate the cytotoxic effects between cancer and normal cells to minimize adverse events and improve clinical outcome. In fact, research on the development of novel, non-toxic therapeutics has been one of the most actively pursued priorities in this area (Guedes et al. 2018).

Lactoferrin (Lf) is a natural iron-binding glycoprotein, a member of the transferrin family that was first isolated from bovine milk (Sorensen and Sorensen 1940). In humans, this glycoprotein is produced by exocrine glands of mucosal epithelium and exists in several biological secretions, including tears, saliva, milk, and is also a component of the secondary granules of neutrophils (Dinauer et al. 2000; Lönnerdal and Iyer 1995; Metz-Boutigue et al. 1984). It has multiple biological activities, often pleiotropic, that are essential for proper development of newborns and protective effects in adults, including antiviral, antibacterial, antiparasitic, and antioxidant properties (Chung et al. 2012; Li et al. 2017; Neville and Zhang 2000; Okubo et al. 2016; Redwan et al. 2016; Safaeian et al. 2015). In addition, this glycoprotein modulates the immune system and regulates the inflammatory responses (Baveye et al. 1999; Kruzel et al. 2017) by influencing myelopoiesis (Sawatzki and Rich 1989; Zimecki et al. 2013) as well as cytokine and chemokine production (Elass et al. 2002; Guillén et al. 2002; Kimber et al. 2002). In fact, Lf is deeply involved in many critical physiological functions, namely immune regulatory and the redox homeostasis (Zimecki et al. 2021).

Importantly, Lf emerges as a promising anticancer agent due to its well-demonstrated anticancer and anti-metastatic activities against a range of human cancers in vitro and in vivomodels (Arias et al. 2017; Cutone et al. 2020b; Gibbons et al. 2015; Zhang et al. 2015). In addition to direct effects on cancer cells, Lf has influence on immune cells. For example, culture of T lymphocytes isolated from cervical cancer patients in the presence of Lf increased expression of zeta-chain in T cells (Frydecka et al. 2002).

However, the majority of research on Lf-induced anticancer effects was conducted using bovine Lf (bLf) (Chea et al. 2019; García-Montoya et al. 2012; Guedes et al. 2018; Jiang and Lönnerdal 2017; Zhang et al. 2015). bLf shares a 69% amino acid sequence homology with human Lf (hLf) (Pierce et al. 1991) and displays a different glycosylation pattern (García-Montoya et al. 2012; Rascón-Cruz et al. 2021). Glycosylation is an important post-translational modification which directly affects both protein structure and biological functions (Marth and Grewal 2008; Ohtsubo and Marth 2006; Shental-Bechor and Levy 2009). Importantly, the oligosaccharide component of glycoprotein is critical for determination of its pharmacological activity and pharmacokinetics as well as immunogenicity and antigenicity (Wormald et al. 2002). For these reasons, bLf does not mimic all the biological roles of hLf and cannot be used for systemic administration in humans (Parc et al. 2017).

Given many beneficial effects and a potential clinical utility of hLf, several forms of recombinant hLf (rhLf) have been produced in different expression systems (Conesa et al. 2010). A few expression systems were expanded to industrial scale to produce rhLf from fungus *Aspergillus niger* (Agennix, Houston, TX, USA), rice (Bioscience, Sacramento, CA, USA) and transgenic cows (Pharming, Leiden, Netherlands) (Conesa et al. 2010). While the primary and secondary structures of most rhLfs are identical with the natural hLf, the glycosylation process associated with each expression system generated a final product that is not fully compatible because of significant alterations in the glycan profile. Several studies have shown anticancer activity of different forms of rhLf expressed in rice (Bezault et al. 1994), fungus *Aspergillus niger* (Xiao et al. 2004) and yeast *Pichia pastoris* (Iglesias-Figueroa et al. 2019). However, rhLfs derived from fungi and yeast expression systems display high levels of mannose N-linked glycan, which may be immunogenic and antigenic, limiting a potential for parenteral therapeutic administrations (Gerngross 2004; Kruzel et al. 2013).

Therefore, we developed a production of a novel rhLf that is compatible with the natural hLf and suitable for parenteral applications, including intravenous and intratumoral injections, or inhalation (Kruzel et al. 2013). Expression of rhLf in Chinese hamster ovary (CHO) cell system allows mammalian glycosylation of the protein generating glycoform of rhLf that is comparable with Lf isolated from human milk (Kruzel et al. 2013), providing optimal structure for hLf pleiotropic activities and safety (biocompatibility) upon its parenteral therapeutic use.

In this study, we investigated the effects of this novel rhLf on human lung adenocarcinoma cell growth and migration as well as its mechanisms of action. We also examined the effect of this rhLf on cytotoxicity in normal human bronchial epithelial (NHBE) cells compared to etoposide. Here, we demonstrate for the first time the selective anticancer activities of this novel rhLf against human lung cancer cells with no cytotoxic effect on NHBE cells. These results provide a potential clinical utility of this rhLf for treatment of patients with lung cancer.

## Materials and Methods

### Recombinant Human Lactoferrin

Human rhLf was expressed in CHO cells and purified as described by Kruzel et al. (2013). It was supplied by PharmaReview Corporation (Houston, Texas, USA) as lyophilized powder (< 15% iron-saturated; < 0.5 endotoxin units mg^–1^) and reconstituted in culture medium to prepare a stock solution at concentration 1 mg/ml before experiments.

### Cell Culture

The human lung adenocarcinoma cell line (A549) was obtained from the European Collection of Cell Cultures (ECACC, cat.no. 86012804). The A549 cells were cultured in DMEM (Biowest, France) containing 10% heat-inactivated fetal bovine serum (FBS, Biowest, France) and 100 units/ml penicillin and 100 μg/ml streptomycin (Biowest, France). NHBE cells (cat. no. C-12640) were purchased from PromoCell and cultured in Airway Epithelial Cell Growth medium (PromoCell, Germany) supplemented with FBS and growth factors. Both cells were cultured in standard cell conditions (37 °C, 5% carbon dioxide and 90% humidity) in an incubator (HeraCell iVios160, ThermoFisher Scientific, USA).

### Cell Viability Assay

Cell viability was determined by performing colorimetric WST-1 assay (Takara, Japan) according to the manufacture’s instruction. To evaluate effect of rhLf on cancer cell growth, A549 cells were seeded in 96-well plate at the density of 5,000 cells per well and cultured for 24 h. The cells (about 50% confluence) were treated with rhLf at different concentrations (0.1, 0.2, 0.5 and 1 mg/ml). The number of live cells was measured after 24 h and 72 h of treatment.

To determine cytotoxicity, NHBE cells were plated in 96-well plate at the density of 10,000 per well. The cells (100% confluence) were treated with rhLf (1 mg/ml) or etoposide (100 µM) (Sigma-Aldrich, USA). The number of viable cells was measured after 72 h of treatment.

At the end of experiments, 10 µl of WST-1 solution was added to each well and the absorption of formazan was measured after 1 h incubation using a microplate reader (iMARK, Bio-Rad) at 450 nm. The number of live cells in response to different treatments was expressed as a percentage of the control cells which constituted 100%.

### Cell Morphology

The influence of rhLf on cell morphology was examined using an inverted microscope with phase contrast (Opta-Tech, software OptaView 7). A549 and NHBE cells were photographed after indicated time of treatment at 100 × magnification.

### Annexin V/PI Assay

Apoptotic cell populations in control and rhLf-treated cells were quantified by a double-staining of cells with Annexin V and propidium iodide (PI) using the AnnexinV/PI apoptosis detection kit (Biolegend, USA) according to the manufacture’s instruction.

A549 cells were seeded in six-well plates at density of 300,000 cells per well and culture for 24 h. Next, cells were treated with rhLf at different concentrations for 24 h and 48 h. Cells were collected and washed with Cell Staining Buffer (Biolegend, USA). Then, cells were stained with Annexin V and PI in Annexin Binding Buffer for 15 min at room temperature in the dark. Apoptotic cells were analyzed by flow cytometry using CytoFlex cytometer (Beckman Coulter, USA). The acquired results were quantified using Kaluza 2.1 software (Beckman Coulter, USA). Cells were gated according to PI and Annexin V staining. Cells positive for Annexin V only were considered as early apoptotic, cells positive for PI only were considered necrotic and cells double positive were taken as late apoptotic cells.

### Caspase-3/7 Activity Assay

The Caspase-Glo 3/7 Assay (Promega, USA) was used to determine cell apoptosis according to the manufacturer’s instruction. A549 cells were plated on 96-well plate at a density of 8000 cells per well. After 24 h culture, cells were treated with rhLf at three different concentrations. Caspase activity was determined after 24 h and 48 h incubation. Luminometer readings were taken one hour after adding the Caspase-Glo 3/7 Reagent to cells at ratio 1:1. Luminescence was recorded on microplate reader (Synergy H1, Bio-Tek, USA) at gain 100. The amount of luminescence produced is proportional to the number of apoptotic cells in the sample.

### Cell Cycle Analysis

A549 cells were seeded in six-well plates at density of 200,000 cells per well and cultured for 24 h. Next, cells were treated with rhLf at indicated concentrations for 24 h. Then, the cells were collected, washed with cold DPBS (Biowest, France) and fixed in 70% ethanol at 4 °C overnight. After washing in DPBS, the cells were incubated with ribonuclease (Sigma-Aldrich, USA) at final concentration of 100 µg/ml at 37 °C for 30 min. Next, PI (Sigma-Aldrich, USA) solution was added to the cells at final concentration of 50 µg/ml prior to analysis. The stained cells were analyzed by flow cytometry using CytoFlex cytometer (Beckman Coulter, USA). Cell cycle distributions were assessed in 20,000 cells collected from each sample and the percentage of cells in each cell cycle phase was calculated.

### Migration Assay

Cancer cell migration was visualized and measured by wound healing assay*.* A549 cells were seeded on 12-well plates at density of 40,000 cells per well and incubated for 24 h. The wound space was scratched with 100 μl pipette tip and cells were rinsed with fresh medium to remove detached cells. Next, cells were treated with rhLf at indicated concentrations. Wound closure in response to treatment was monitored under inverted microscope (Opta-Tech, Poland) and photographed to assess cell migration at time intervals of 0 h, 24 h and 48 h. Images were analyzed by Image J software by measuring of the width of the scratch area at different time intervals to calculate wound closure. The area of wound at 0 h time point was expressed as a 100%.

### Statistical Analyses

All results were expressed as mean ± standard deviation (SD). Data were obtained from at least three separate experiments. Statistical analysis was performed using Statistica 13.3 software (StatSoft). The normal distribution of continuous variables was verified with the Shapiro–Wilk test. Statistical comparisons for all variables with a normal distribution were made using paired *t* test. The variables with non-normal distributions were compared using the Wilcoxon signed rank test. A level of *p* less than 0.05 was considered statistically significant (*p < 0.05).

## Results

### rhLf Inhibits Lung Cancer Cell Growth

**Fig. 1:**
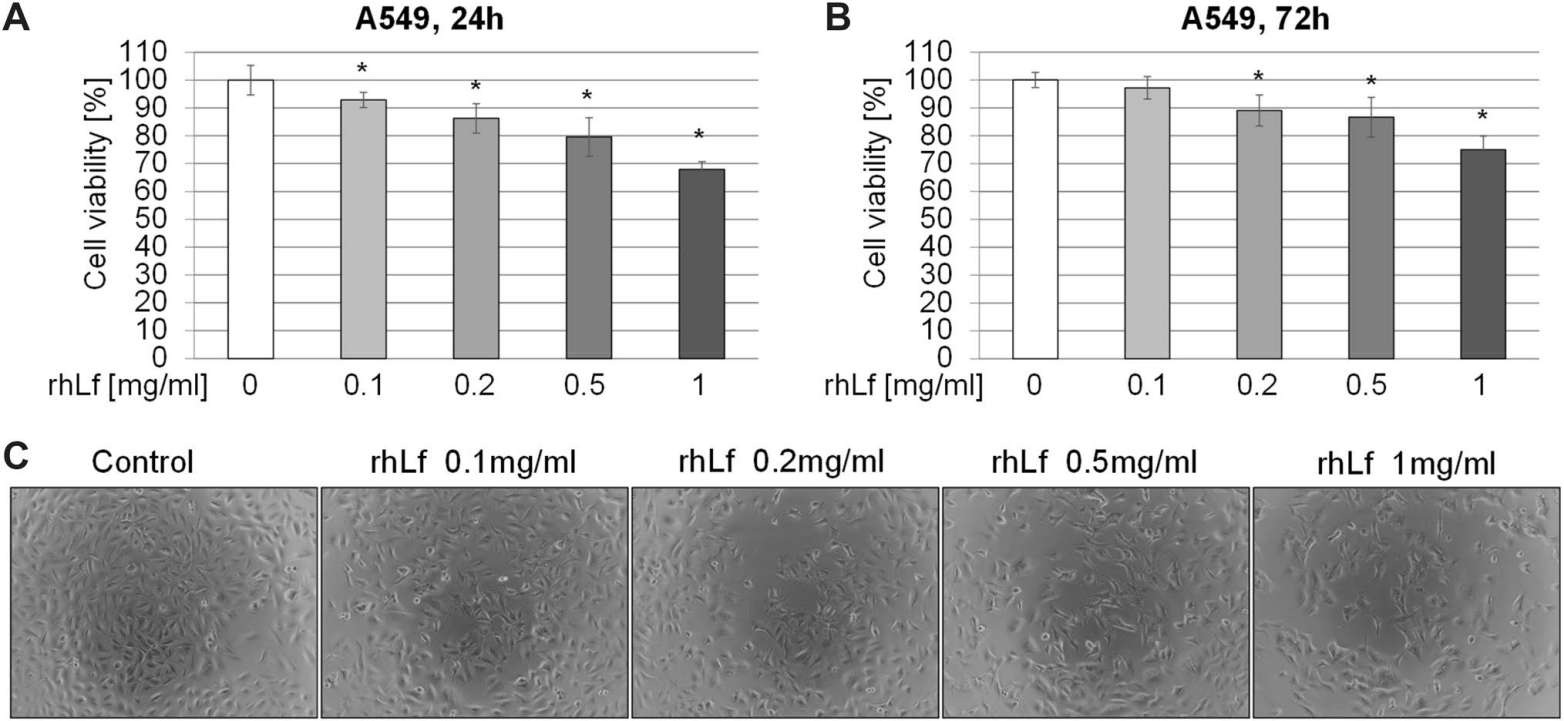
rhLf inhibits human lung cancer cell growth. A 549 cells were cultured in the presence of rhLf at indicated concentrations for 24 h (**A**) or 72 h (**B**) and the number of live cells was quantified by a WST assay. The results were calculated as a percentage of the control cells and expressed as mean ± S.D. of *n* = 6. *p < 0.05 compared to the control (0). Visualization of the effect of rhLf on cancer cell growth and morphology (**C**). Representative phase-contrast cell images are shown after 24 h of treatment (100 × magnification)

First, we examined the effect of rhLf on human lung adenocarcinoma cell growth. A549 cells were treated with four different concentrations of rhLf at the range 0.1–1 mg/ml and cancer cell growth was monitored for 72 h. The number of live cells was determined by WST assay. The results showed that rhLf induced a concentration-dependent inhibition of A549 cell growth after 24 h treatment (Fig. 1A) and this anticancer effect was observed for the next 72 h (Fig. 1B). For example, cell exposure to 1 mg/ml of rhLf for 24 h and 72 h significantly decreased lung cancer cell growth by 32.1 ± 2.8% (*p* = 0.0001) and 25.0 ± 4.9% (*p* = 0.0024), respectively (Fig. 1A and B). In addition, the cancer cell images after 24 h treatment visualized the lower density of cells cultured in the presence of rhLf as compared to cell density in the control (Fig. 1C).

### rhLf Induces Apoptosis in Lung Cancer Cells

**Fig. 2:**
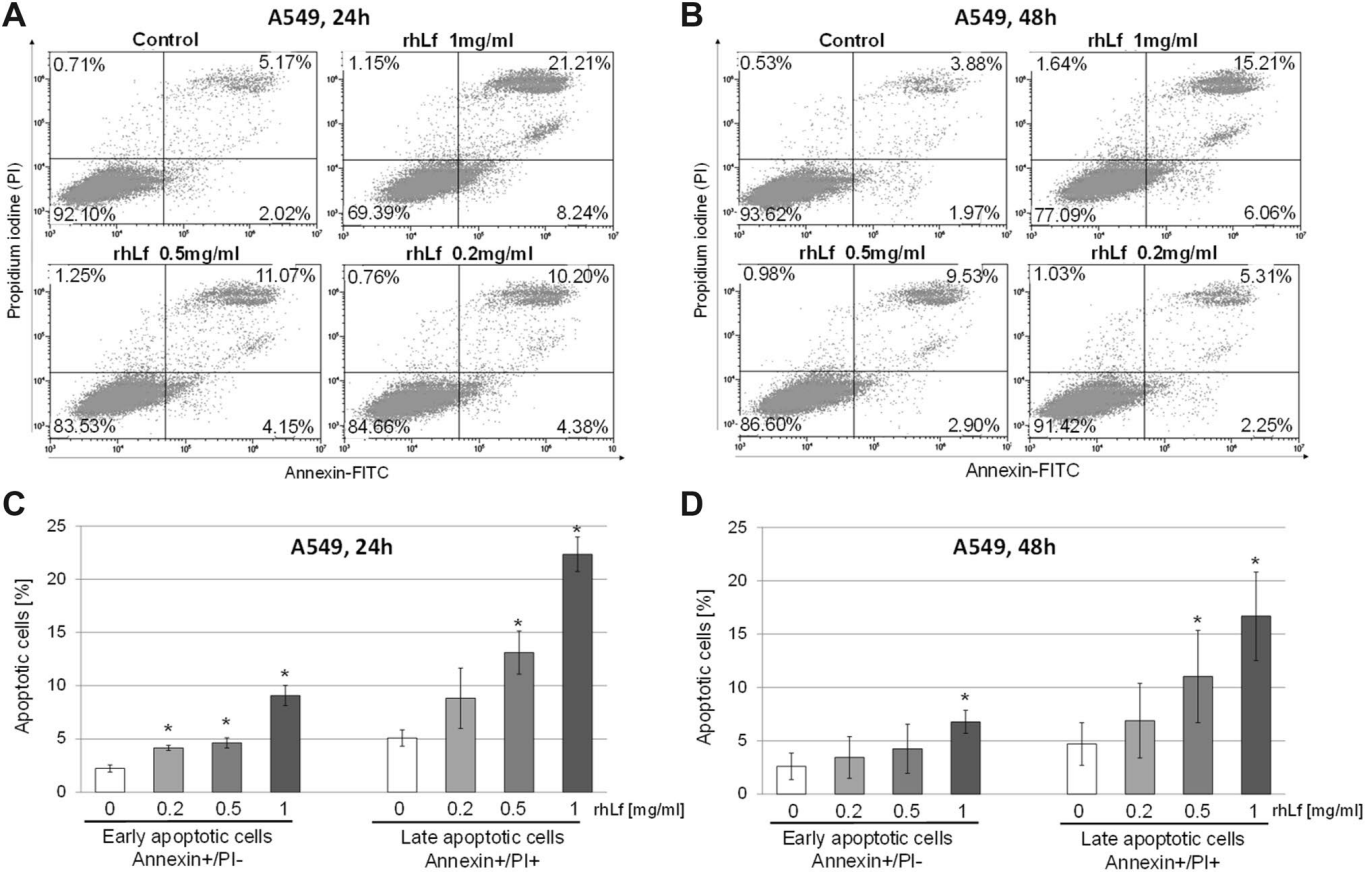
rhLf induces apoptosis in lung cancer cells. Representative plots of flow cytometry analysis of Annexin V and PI staining of the control and cells treated with rhLf at indicated concentrations for 24 h (**A**) or 48 h (**B**). Cell populations in four quadrants: viable cells (Annexin^–^/PI^–^), early apoptotic cells (Annexin^+^/PI^–^), late apoptotic cells (Annexin^+^/PI^+^), necrotic cells (Annexin^–^/PI^+^). Quantification of early and late apoptotic cells in response to rhLf treatments after 24 h (**C**) and 48 h (**D**). Values represent mean ± S.D. of *n* = 3. *p < 0.05 compared to the control (0)

The inhibition of cancer cell growth may result in initiation of apoptosis process. Therefore, to evaluate whether the suppression of A549 cell growth by rhLf is associated with an induction of apoptosis, we detected apoptotic cells by Annexin V and PI staining. The representative cytograms showed changes in the percentage of live (Annexin^–^/PI^–^), early apoptotic (Annexin^+^/PI^–^), late apoptotic (Annexin^+^/PI^+^) and necrotic cells (Annexin^–^/PI^+^) in response to rhLf treatment at indicated concentrations after 24 h (Fig. 2A) and 48 h (Fig. 2B). The quantitative analysis illustrated a concentration-dependent increase in the percentage of early and late apoptotic cells in A549 cells cultured in the presence of rhLf as compared to the control (Fig. 2C and D). For example, the 24 h exposure of A549 cells to 1 mg/ml of rhLf increased 4.5-fold the percentage of early apoptotic cells (9.1 ± 0.9% vs control 2.2 ± 0.3%, *p* = 0.0028) and 4.3-fold the percentage of late apoptotic cells (22.3 ± 1.6% vs control 5.1 ± 0.8%, *p* = 0.0014) (Fig. 2C). Similarly, we detected about 2.5- and 3.5-fold higher the percentage of early and late apoptotic cells in A549 cells treated with 1 mg/ml of rhLF for 48 h (6.8 ± 1.1 vs control 2.6 ± 1.3%, *p* = 0.0011 and 16.7 ± 4.2% vs control 4.7 ± 2.0%, *p* = 0.011, respectively) (Fig. 2D).

**Fig. 3:**
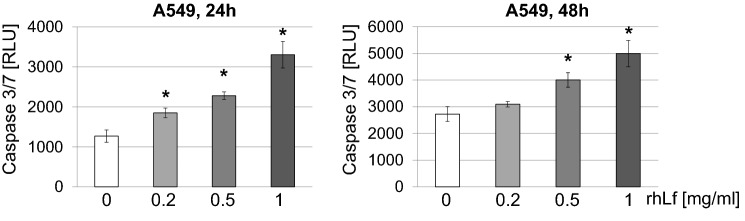
rhLf increases activity of caspase-3 in lung cancer cells. A549 cells were treated with rhLf at indicated concentrations. The levels of caspase-3/7 activity in cells were measured as a luminescence light unit after 24 and 48 h of treatment. Data are expressed as relative luminescence units (RLU), mean ± S.D. of *n* = 3. *p < 0.05 compared to the control (0)

Caspase-3 is a well-known downstream executor of the apoptotic pathway, through its ability to cleave several cellular substrates. Therefore, to evaluate whether rhLf-induced an increase in apoptotic cells is associated with activation of this enzyme, we measured the caspase-3 activity. As shown in Fig. 3, the treatment of A549 cells with rhLf for 24 h resulted in a significant increase in the activation of caspase-3 in a concentration-dependent manner. We observed about threefold higher levels of caspase activity in A549 cells exposed to 1 mg/ml of rhLf as compared to the control cells (Fig. 3). The significantly elevated levels of caspase-3 activity were also detected in cancer cells exposed to rhLf at concentrations of 0.5 mg/ml and 1 mg/ml after 48 h of treatment (Fig. 3). Overall, the data demonstrated that rhLf induced apoptosis in human lung adenocarcinoma cells by activation of caspase-3.

### rhLf Alters Cell Cycle of Lung Cancer Cells

**Fig. 4:**
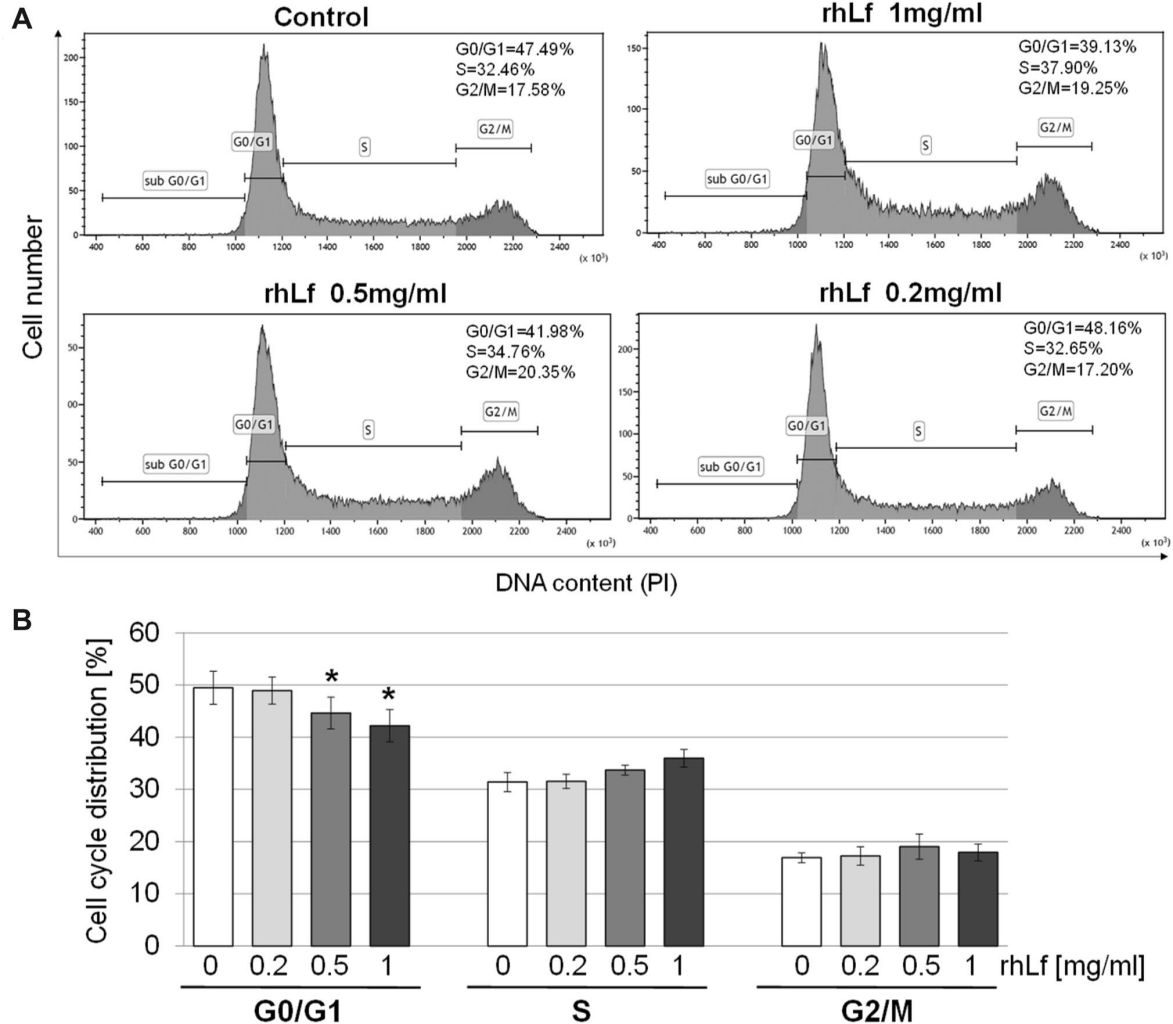
rhLf alters lung cancer cell cycle progression. A549 cells were treated with rhLf at indicated concentrations for 24 h. DNA content was assessed by flow cytometry analysis of cells stained with PI. Representative histograms of the cell cycle distribution at G0/1, S and G2/M phase in the control and cells treated with rhLf are shown (**A**). Bar graph shows the percentage of cells at each phase of cell cycle in the control cells (0) and cells treated with rhLf at indicated concentrations (**B**). Data are expressed as mean ± S.D. of *n* = 3. *p < 0.05 compared to the control (0)

To better understand the mechanisms responsible for rhLf-induced cancer cell growth suppression, we also investigated the effect of rhLf on cancer cell cycle distribution. Separation of A549 cells in G0/G1, S and G2/M phase was performed by flow cytometry analysis based on fluorescence intensity of cells stained with PI. Figure 4A shows representative profiles of cell cycle distribution in the control cells and cells exposed to rhLf at indicated concentrations for 24 h. When A549 cells were treated with 1 mg/ml and 0.5 mg/ml of rhLf, we observed a significant decrease in the percentage of cells at G0/G1 phase, with a corresponding increase in the percentage of cells at S phase. For example, with respect to the control cells, the cell population in the G0/G1 phase decreased from 49.5 ± 3.2 to 42.2 ± 3.1% (*p* = 0.0133) in the presence of 1 mg/ml rhLf (Fig. 4B). This was associated with increased percentage of cells in the S phase from 31.4 ± 1.8 to 36.0 ± 1.7% (*p* = 0.0524) in rhLf-treated cells as compared to the control (Fig. 4B). The results suggested that rhLf induced growth arrest of lung cancer cells at the S phase.

### rhLf Inhibits Cancer Cell Migration

**Fig. 5:**
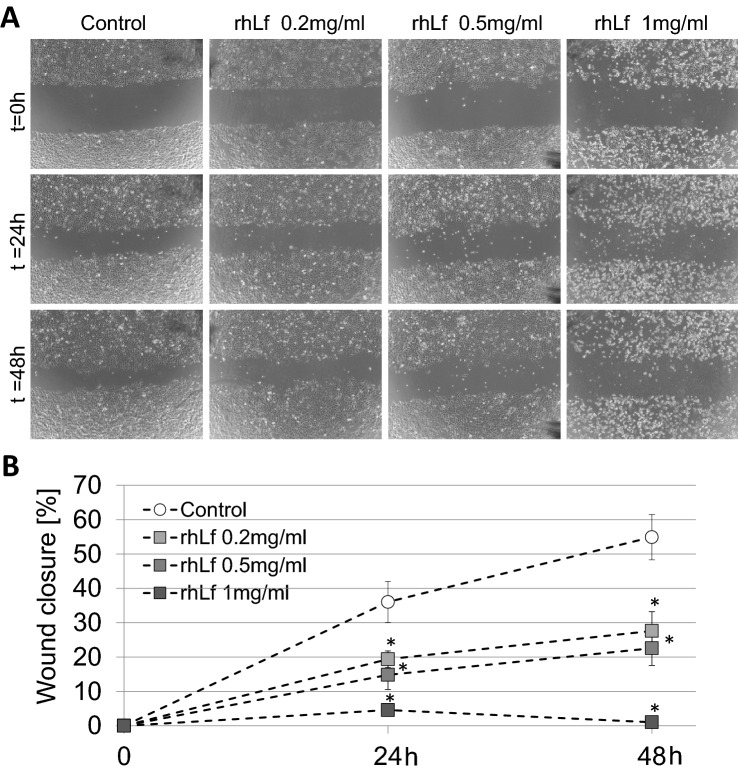
rhLf attenuates lung cancer cell migration. Cell migration was evaluated by a wound healing assay. Confluent of A549 cells was wounded and treated with or without rhLf at indicated concentrations (**A**). Cells were photographed at time zero (*t* = 0 h), after 24 h (*t* = 24 h) and after 48 h (*t* = 48 h). The graph represents the percentage of wound closure during 48 h of cell exposure to different concentrations of rhLf (B). Data are expressed as mean ± S.D. of *n* = 4. *p < 0.05 compared to the control

Metastasis is the primary cause of mortality in most cancer patients. Therefore, the repression of the metastatic process has been an important strategy in cancer therapy. Since cell migration is an important process involved in the metastasis formation (Valster et al. 2005), we performed a wound healing assay to evaluate the effect of rhLf on cancer cell migration. Figure 5A displays the representative images of the wound area photographed at 0 h, 24 h and 48 h after treatment at indicated concentrations of rhLf. The quantitative analysis of wound closure in the control and rhLf-treated cells showed that treatment of A549 cells with rhLf resulted in a significant attenuation of cancer cell migration in all tested concentrations (Fig. 5B). For example, the calculated wound closure was 54.9 ± 6.6% in the control cells after 48 h, where in rhLf-treated cells at 0.2 mg/ml and 0.5 mg/ml was only 27.6 ± 5.6% (*p* = 0.0017) and 22.6 ± 5.0% (*p* = 0.0019), respectively (Fig. 5B). Moreover, exposure of A549 cells to 1 mg/ml of rhLf almost completely inhibited cell migration (Fig. 5A and B). These results demonstrated that rhLf was effective in attenuation of lung cancer cell migration.

### rhLf Has No Cytotoxic Effect on NHBE Cells

**Fig. 6:**
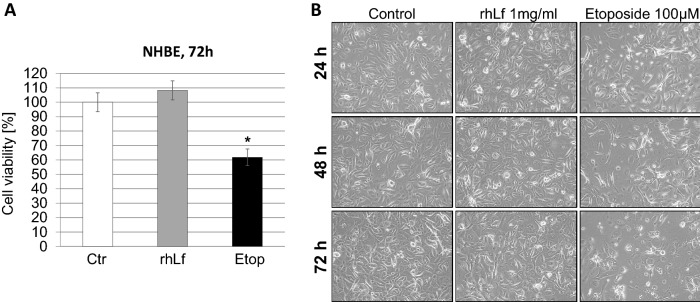
rhLf does not induce cytotoxicity in NHBE cells. Confluent of NHBE cells was exposed to the highest tested concentration of rhLf (1 mg/ml) or etoposide (100 µM) (Etop). Cell viability was quantified by a WST-1 assay after 72 h (**A**). Data are expressed as mean ± S.D. of *n* = 3. *p < 0.05 compared to the control (Ctr). Visualization of the effect of rhLf and etoposide on the morphology and viability of NHBE cells (**B**). Representative phase-contrast cell images are shown after 24 h, 48 h and 72 h of treatment (100 × magnification)

In general, the effectivity of chemotherapy in patients is restricted by its inevitable side effects. Therefore, it was an important to evaluate whether rhLf has a cytotoxic effect on normal human lung cells. Confluent of NHBE cells was cultured in the presence of rhLf at the highest tested concentration and monitored for 72 h. The results showed that the viability of NHBE cells exposed to 1 mg/ml of rhLf was comparable to the control (Fig. 6A). In addition, the NHBE cell images visualized confluent of healthy epithelial cells with cell–cell junctions in the presence of rhLf similar to confluent of the control cells (Fig. 6B). In contrast, exposure of NHBE cells to 100 μM of etoposide significantly decreased the cell viability by 38.2 ± 10.1% (*p* = 0.0164) (Fig. 6A). Moreover, NHBE cell images after 24 h, 48 h and 72 h treatment with etoposide showed profound morphological changes characterized by disruption of epithelial cell confluent, completely loss of cell–cell contact, and overall decrease in the number of attached cells as compared to confluent cells observed in the control and rhLf-treated cells (Fig. 6B).

Together, the data indicated that this novel rhLf did not affect the viability of normal human epithelial cells at the concentration that induced the anticancer effects in human lung cancer cells.

## Discussion

Here, we aimed to ascertain the anticancer activity of a novel rhLf that displays glycosylation profile comparable with the natural hLf, against human lung adenocarcinoma cells. Our study demonstrated that rhLf exhibits the anticancer effects in human lung adenocarcinoma cells and has no cytotoxic effect on NHBE cells. These findings are clinically significant because demonstrate a therapeutic potential of this CHO-derived rhLf for parenteral applications, including intratumoral and intravenous injections in patients with lung cancer.

Malignancy has become a major public health issue, with an estimated 19.3 million new cases and almost 10 million cancer deaths worldwide occurred in 2020 (Ferlay 2020; Sung et al. 2021). Lung cancer is still the leading cause of cancer death. Non-small cell lung cancer (NSCLC) becomes the most common subtype, accounting for approximately 85% of cases. Lung adenocarcinoma is the most prevalent and aggressive form of NSCLC and its frequency of occurrence is increasing rapidly (Duma et al. 2019; Lu et al. 2019). Over the past several years, targeted therapy and immune checkpoint inhibitors have been revolutionized cancer treatment. However, despite these advancements in clinical care, the majority of patients do not respond to these therapies or develop acquired resistance (Qiao et al. 2018; Wang et al. 2020; Yoneda et al. 2018)*.* Although, they are generally well tolerated, but they are not without toxicities (Bertrand et al. 2015; Remon et al. 2018; Wang et al. 2017). Thus, standard chemotherapy is the only option for the most patients with advanced lung cancer (Carbone et al. 2015). Overall, the main limitation of standard chemotherapy is profound side effects resulted in drug-induced cytotoxicity on normal cells (Carbone et al. 2015).

Therefore, the major therapeutic challenge has been to develop more effective treatments that discriminate between normal and cancer cells to minimize adverse events. For this reason, the use of non-toxic natural products has been one of the current strategies for cancer therapy.

Lf, a natural iron-binding protein, has attracted scientific interest because of its immunomodulatory and pleiotropic functions (Kane et al. 2003; Kruzel et al. 2017; Legrand et al. 2006), including anticancer activities (Bezault et al. 1994; Kirkpatrick et al. 1971). Some studies indicate that the iron-binding ability of Lf as well as the interaction between Lf and its specific receptors expressed on different cells are responsible for the diverse biological properties (Adlerova et al. 2008). Depending on its iron content, Lf possesses two opposite conformational states, the open iron-free form (apo-Lf) and the closed iron-binding form (holo-Lf) (Baker and Baker 2012). The native form of Lf, produced in physiological conditions, has low iron saturation rate between 10 and 20%, leading to the prevalence of apo- and monoferric Lf. In contrast, the holo-form is mostly present in inflammation and infection sites characterized by high levels of free iron (Rosa et al. 2018). It was demonstrated that cancer cells demand higher concentrations of iron to grow and over-express the genes implicated in iron uptake (Kazan et al. 2017). Lf is able to inhibit cancer cell growth by scavenging free iron and decreasing the availability of iron to cancer cells. Additionally, Lf could be implicated in an iron-related mechanism of cancer cell death via ferroptosis (Dixon et al. 2012; Yang et al. 2014). Moreover, depending on the iron saturation rate, Lf can exert dissimilar functions by activating specific signaling pathways (Cutone et al. 2020a; Jiang and Lönnerdal 2012). In this respect, holo-bLf has been demonstrated to be more efficient in inhibition of glioblastoma cell migration than the native bLf. This effect was correlated with higher internalization of iron-saturated bLf as compared with the native form (Cutone et al. 2020a).

Lf is a highly glycosylated protein. Glycosylation plays a critical role in determining protein structure, biological function, stability and influence immunogenicity and antigenicity (Almond et al. 2013; Barboza et al. 2012; Wormald et al. 2002). hLf has a lot of sialylated structures and a high abundance of fucosylated oligosaccharides, and no significant amounts of high-mannose structures (Parc et al. 2017). While bLf is commercially available, and used as a dietary supplement, it is not applicable for parenteral administrations in humans due to high risk of antigenic incompatibility. rhLf has been expressed in different cell types (BHK cells, Sf9 insect cells), plants (rice, fungi, yeast), and transgenic animals (cow, goat, sheep, mouse) (Choi et al. 2008; Nandi et al. 2005; Ward et al. 1995, 1997; Yang et al. 2008; Zhao et al. 2006). Since protein glycosylation process is a species-specific and tissue-specific modification system, rhLf may present some glycan patterns that are typical of glycome of a species that it was expressed. Despite the high amino acid sequence homology and structural similarity between rhLf and hLf, each form of rhLf exhibits a unique glycosylation pattern that is not compatible with the natural hLf isolated from human milk. The anticancer effects of rhLf were examined using different rhLf expressed in yeast *Pichia pastoris* (Iglesias-Figueroa et al. 2019), fungus *Aspergillus niger* (Hayes et al. 2006, 2010; Jonasch et al. 2008; Xiao et al. 2004), and rice (Bezault et al. 1994). Glycan analysis showed that the expression of rhLf in yeast led to non-sialylated protein. Moreover, no fucosylation was detected for any glycan and high-mannose structure was observed (Choi et al. 2008). Similarly, the expression of rhLf in *Aspergillus* resulted in highly mannosylated glycoproteins that are typical for fungi (Gerngross 2004). rhLf derived from rice also lacks sialic acid and contains xylose, which is consistent with plant post-translational modification patterns (Matsumoto et al. 1995). Therefore, none of these forms of rhLf that showed anticancer effects can be used for parenteral therapy in humans due to a lack of adequate glycosylation profile.

To address this problem, we have established production of a novel rhLf that was expressed in CHO cells. This expression system has the advantage of incorporation mammalian type glycosylation in the recombinant protein. The mass spectrometry analysis of this new rhLf showed N-linked fucosylated and sialylated glycans at two glycosylation sites that are comparable with the natural hLf (Kruzel et al. 2013). We previously have shown that this rhLf modulates gene expression profile in blood cells (Kruzel et al. 2021) and displays antioxidant and antibacterial functions (Kruzel et al. 2013).

However, anticancer effects of this novel rhLf are unknown. Therefore, in the present study, the new rhLf was examined for its anticancer activity in A549 cells obtained from human lung adenocarcinoma that represents the most prevalent and aggressive subtype of NSCLC. The results showed that rhLf inhibits cancer growth in a concentration-dependent manner. The highest tested concentration of rhLf (1 mg/ml) inhibited cancer cell growth by about 32% and 25% after 24 h and 72 h treatment, respectively. Previous study has reported that bLf inhibited A549 cell growth (Tung et al. 2013). Moreover, our results showed the efficacy of rhLf was comparable to bLf in A549 cells.

Induction of apoptosis is one of the fundamental mechanisms that impede cancer growth and proliferation. To evaluate whether rhLf inhibited cancer cell growth by induction of apoptosis, we performed the double staining of A549 cells with Annexin V-FITC and PI to detect early and late apoptotic cells, and necrotic cells. Flow cytometry analysis showed that 1 mg/ml of rhLf increased over fourfold the percentage of early and late apoptotic cells after 24-h exposure. Previous studies have demonstrated that bLf induced apoptosis by phosphatidylserine (PS) externalization in prostate PC-3, osteosarcoma MG-63, breast cancer MDA-MB-231, and oral squamous cell carcinoma (Chea et al. 2019; Guedes et al. 2018; Pereira et al. 2016; Zhang et al. 2015). Recently, it was reported that rhLf expressed in yeast *Pichia pastoris* activated apoptosis via PS externalization in breast cancer cells (Iglesias-Figueroa et al. 2019). Our study also demonstrated externalization of PS in human lung cancer cells in response to rhLf derived from CHO cells. To further evaluate the mechanism of rhLf-induced cancer cell death, we measured the levels of caspase-3 activity. Caspases play the key role in the initiation and execution of programmed cell death. Caspase-3 is a central executioner of apoptosis and is responsible for the proteolytic degradation of structural and signaling proteins that ultimately results in cell death (Ji et al. 2011). We found that treatment of A549 cells with rhLf resulted in a significant increase in the levels of caspase-3 activity in a concentration-dependent manner after 24 h. Together, our data showed rhLf-induced PS externalization was concomitant with increased the levels of caspase-3 activity in lung cancer cells.

Cell cycle arrest is an important signal for inhibition of cell proliferation (Qiu et al. 2013). Moreover, the induction of apoptosis might be mediated through cell cycle arrest (Goodell et al. 2008; Mantena et al. 2006). To determine whether rhLf inhibited cancer cell growth as a result of induction of cell cycle arrest, we examined the effect of rhLf on the cell cycle profile. Flow cytometry analysis suggested that rhLf induced A549 cell growth arrest at S phase of cell cycle. It was reported that rhLf expressed in fungus *Aspergillus* induced growth arrest at G1 phase in head and neck cancer cells (Xiao et al. 2004). Moreover, bLf exerted anticancer effects by cell cycle arrest at G1 or G2 phase depends on cancer cell types and concentrations (Zhang et al. 2014). For example, this study showed that bLf caused cell cycle arrest of MDA-MB-231 cells at G2 phase (Zhang et al. 2014). For comparison, treatment of MDA-MB-231 cells with rhLf derived from yeast resulted in the cell cycle arrest at S phase (Iglesias-Figueroa et al. 2019). This suggests that inhibition of MDA-MB-231 cell growth in response to rhLf and bLf may be mediated by different molecular mechanisms. The cell cycle analysis of rhLf-treated breast cancer cells showed reduction of the percentage of cells at G0/G1 phase that was accompanied by increment cells in S phase (Iglesias-Figueroa et al. 2019). Interestingly, A549 cells exposed to rhLf from CHO cells resembled the cell cycle arrest profile that was observed in these breast cancer cells treated with rhLf derived from yeast.

The increased migration ability of cancer cells plays an important role in tumor invasiveness and metastasis. Lung cancer cells have highly invasive and metastatic properties with an ability to migrate into surrounding tissues (Collins et al. 2007). It has been reported that bLf diminished migration of breast cancer cells (Duarte et al. 2011). We also tested the effect of rhLf on migration of lung cancer cells. The exposure of A549 cells to rhLf resulted in a significant attenuation of cancer cell migration in a concentration-dependent manner. Therefore, inhibition of cancer cell migration is an important aspect of anticancer activity of potential drugs, in addition to inhibition of cell proliferation and induction of apoptosis. Finding novel biotherapeutics that specifically exert cytotoxicity in cancer cells, with little or no activity on normal cells is highly desirable. To evaluate whether rhLf possesses selective cytotoxicity against lung cancer cells, we also examined its cytotoxicity in NHBE cells. We found that exposure of NHBE cells to the highest tested concentration of rhLf showed a similar cell viability and morphology that was detected in the control cells. Most of chemotherapeutic agents induce cytotoxicity in cancer cells, but they usually do not discriminate between normal and cancer cells. Etoposide is telomerase II inhibitor that is used in chemotherapy for lung cancer (Datta and Sinha 2019; Liang et al. 2017). Thus, NHBE cells were treated with etoposide as positive control. In contrast to rhLf, etoposide induced cytotoxicity and decreased viability of NHBE cells by 40%. The selective cytotoxicity of rhLf from yeast was also demonstrated in breast cancer cells in comparison with non-cancerous breast epithelial cells (Iglesias-Figueroa et al. 2019). Similarly, bLf induced specific cytotoxicity in oral squamous cell carcinoma in contrast to normal human oral keratinocytes (Chea et al. 2019). The direct recognition of cancer cells and selection between cancerous and normal cells by Lf may involve a primary interaction with cancer cell surface receptors. Most cancerous cells have a high content of proteoglycans, glycosaminoglycans and sialic acids, and all these molecules have been shown to interact with Lfs (Damiens et al. 1998; Legrand et al. 2006). Binding of Lf to these specific receptors results in activation of different signaling pathways that may induce cancer cell apoptosis, cell cycle arrest or inhibition of migration (González-Chávez et al. 2009; Rodrigues et al. 2009). Thus, anticancer specificity and selectivity of Lfs could be mediated by recognition of these receptors. The lack of cytotoxicity of this novel rhLf in normal human lung cells is very important for a potential development of the biotherapeutic for treatment of lung cancer.

## Conclusion

Biotherapeutics containing glycoprotein must possess glycosylation pattern comparable with natural protein to avoid potential immunogenicity and antigenicity during parenteral applications. This study showed the novel rhLf that resembles glycan structure of the natural hLf, exhibited the anticancer effects in human lung adenocarcinoma cells. Importantly, our results also demonstrated that this rhLf has no cytotoxic effects on normal human lung epithelial cells. The ability to specifically target cancer cells is one of the desired properties of an ideal anticancer drug. This unique rhLf provides a new opportunity for intratumoral injections, allowing for direct interaction of rhLf with cancer cells and tumor-infiltrating immune cells. Further preclinical study is needed to fully evaluate the efficacy and the precise mechanisms of this rhLf on inhibition of tumor growth.

## Data Availability

All data generated and analyzed in this study are available upon request from the first author.

## References

[CR1] Adlerova L, Bartoskova A, Faldyna M (2008). Lactoferrin: a review. Veterinarní Medicína.

[CR2] Almond RJ, Flanagan BF, Antonopoulos A (2013). Differential immunogenicity and allergenicity of native and recombinant human lactoferrins: role of glycosylation. Eur J Immunol.

[CR3] Arias M, Hilchie AL, Haney EF (2017). Anticancer activities of bovine and human lactoferricin-derived peptides. Biochem Cell Biol.

[CR4] Baker HM, Baker EN (2012). A structural perspective on lactoferrin function. Biochem Cell Biol.

[CR5] Barboza M, Pinzon J, Wickramasinghe S (2012). Glycosylation of human milk lactoferrin exhibits dynamic changes during early lactation enhancing its role in pathogenic bacteria-host interactions. Mol Cell Proteomics.

[CR6] Baveye S, Elass E, Mazurier J (1999). Lactoferrin: a multifunctional glycoprotein involved in the modulation of the inflammatory process. Clin Chem Lab Med.

[CR7] Bertrand A, Kostine M, Barnetche T (2015). Immune related adverse events associated with anti-CTLA-4 antibodies: systematic review and meta-analysis. BMC Med.

[CR8] Bezault J, Ramesh B, Wiprovnick J (1994). Human lactoferrin inhibits growth of solid tumors and development of experimental metastases in mice. Cancer Res.

[CR9] Bray F, Ferlay J, Soerjomataram I (2018). Global cancer statistics 2018: GLOBOCAN estimates of incidence and mortality worldwide for 36 cancers in 185 countries. CA Cancer J Clin.

[CR10] Carbone DP, Gandara DR, Antonia SJ (2015). Non-small-cell lung cancer: role of the immune system and potential for immunotherapy. J Thorac Oncol.

[CR11] Chea C, Haing S, Miyauchi M (2019). Molecular mechanisms underlying the inhibitory effects of bovine lactoferrin on osteosarcoma. Biochem Biophys Res Commun.

[CR12] Choi BK, Actor JK, Rios S (2008). Recombinant human lactoferrin expressed in glycoengineered Pichia pastoris: effect of terminal N-acetylneuraminic acid on in vitro secondary humoral immune response. Glycoconj J.

[CR13] Chung SH, Kang HB, Kim JW (2012). The biological effects of bovine lactoferrin on inflammatory cytokine expression in the PMA stimulated cells. Food Sci Anim Resour.

[CR14] Collins LG, Haines C, Perkel R (2007). Lung cancer: diagnosis and management. Am Fam Physician.

[CR15] Conesa C, Calvo M, Sánchez L (2010). Recombinant human lactoferrin: a valuable protein for pharmaceutical products and functional foods. Biotechnol Adv.

[CR16] Cutone A, Colella B, Pagliaro A (2020). Native and iron-saturated bovine lactoferrin differently hinder migration in a model of human glioblastoma by reverting epithelial-to-mesenchymal transition-like process and inhibiting interleukin-6/STAT3 axis. Cell Signal.

[CR17] Cutone A, Rosa L, Ianiro G (2020). Lactoferrin’s anti-cancer properties: safety, selectivity, and wide range of action. Biomolecules.

[CR18] Damiens E, El Yazidi I, Mazurier J (1998). Role of heparan sulphate proteoglycans in the regulation of human lactoferrin binding and activity in the MDA-MB-231 breast cancer cell line. Eur J Cell Biol.

[CR19] Datta S, Sinha D (2019). EGCG maintained Nrf2-mediated redox homeostasis and minimized etoposide resistance in lung cancer cells. J Funct Foods.

[CR20] Dinauer MC, Lekstrom-Himes JA, Dale DC (2000). Inherited neutrophil disorders: molecular basis and new therapies. Hematology Am Soc Hematol Educ Program.

[CR21] Dixon SJ, Lemberg KM, Lamprecht MR (2012). Ferroptosis: an iron-dependent form of nonapoptotic cell death. Cell.

[CR22] Duarte DC, Nicolau A, Teixeira JA (2011). The effect of bovine milk lactoferrin on human breast cancer cell lines. J Dairy Sci.

[CR23] Duma N, Santana-Davila R, Molina JR (2019). Non-small cell lung cancer: epidemiology, screening, diagnosis, and treatment. Mayo Clin Proc.

[CR24] Elass E, Masson M, Mazurier J (2002). Lactoferrin inhibits the lipopolysaccharide-induced expression and proteoglycan-binding ability of interleukin-8 in human endothelial cells. Infect Immun.

[CR25] Ferlay J (2020) Cancer today. http://gco.iarc.fr/today/home. Accessed 31 May 2021

[CR26] Frydecka I, Zimecki M, Bocko D (2002). Lactoferrin-induced up-regulation of zeta (zeta) chain expression in peripheral blood T lymphocytes from cervical cancer patients. Anticancer Res.

[CR27] García-Montoya IA, Cendón TS, Arévalo-Gallegos S (2012). Lactoferrin a multiple bioactive protein: an overview. Biochim Biophys Acta.

[CR28] Gerngross TU (2004). Advances in the production of human therapeutic proteins in yeasts and filamentous fungi. Nat Biotechnol.

[CR29] Gibbons JA, Kanwar JR, Kanwar RK (2015). Iron-free and iron-saturated bovine lactoferrin inhibit survivin expression and differentially modulate apoptosis in breast cancer. BMC Cancer.

[CR30] González-Chávez SA, Arévalo-Gallegos S, Rascón-Cruz Q (2009). Lactoferrin: structure, function and applications. Int J Antimicrob Agents.

[CR31] Goodell JR, Ougolkov AV, Hiasa H (2008). Acridine-based agents with topoisomerase II activity inhibit pancreatic cancer cell proliferation and induce apoptosis. J Med Chem.

[CR32] Guedes JP, Pereira CS, Rodrigues LR (2018). Bovine milk lactoferrin selectively kills highly metastatic prostate cancer PC-3 and osteosarcoma MG-63 cells in vitro. Front Oncol.

[CR33] Guillén C, McInnes IB, Vaughan DM (2002). Enhanced Th1 response to Staphylococcus aureus infection in human lactoferrin-transgenic mice. J Immunol.

[CR34] Hayes TG, Falchook GF, Varadhachary GR (2006). Phase I trial of oral talactoferrin alfa in refractory solid tumors. Invest New Drugs.

[CR35] Hayes TG, Falchook GS, Varadhachary A (2010). Phase IB trial of oral talactoferrin in the treatment of patients with metastatic solid tumors. Invest New Drugs.

[CR36] Iglesias-Figueroa BF, Siqueiros-Cendón TS, Gutierrez DA (2019). Recombinant human lactoferrin induces apoptosis, disruption of F-actin structure and cell cycle arrest with selective cytotoxicity on human triple negative breast cancer cells. Apoptosis.

[CR37] Ji YB, Qu ZY, Zou X (2011). Juglone-induced apoptosis in human gastric cancer SGC-7901 cells via the mitochondrial pathway. Exp Toxicol Pathol.

[CR38] Jiang R, Lönnerdal B (2012). Apo- and holo-lactoferrin stimulate proliferation of mouse crypt cells but through different cellular signaling pathways. Int J Biochem Cell Biol.

[CR39] Jiang R, Lönnerdal B (2017). Bovine lactoferrin and lactoferricin exert antitumor activities on human colorectal cancer cells (HT-29) by activating various signaling pathways. Biochem Cell Biol.

[CR40] Jonasch E, Stadler WM, Bukowski RM (2008). Phase 2 trial of talactoferrin in previously treated patients with metastatic renal cell carcinoma. Cancer.

[CR41] Kane SV, Sandborn WJ, Rufo PA (2003). Fecal lactoferrin is a sensitive and specific marker in identifying intestinal inflammation. Am J Gastroenterol.

[CR42] Kazan HH, Urfali-Mamatoglu C, Gunduz U (2017). Iron metabolism and drug resistance in cancer. Biometals.

[CR43] Kimber I, Cumberbatch M, Dearman RJ (2002). Lactoferrin: influences on Langerhans cells, epidermal cytokines, and cutaneous inflammation. Biochem Cell Biol.

[CR44] Kirkpatrick CH, Green I, Rich RR (1971). Inhibition of growth of Candida albicans by iron-unsaturated lactoferrin: relation to host-defense mechanisms in chronic mucocutaneous candidiasis. J Infect Dis.

[CR45] Kruzel ML, Actor JK, Zimecki M (2013). Novel recombinant human lactoferrin: differential activation of oxidative stress related gene expression. J Biotechnol.

[CR46] Kruzel ML, Zimecki M, Actor JK (2017). Lactoferrin in a context of inflammation-induced pathology. Front Immunol.

[CR47] Kruzel ML, Olszewska P, Pazdrak B (2021). New insights into the systemic effects of oral lactoferrin: transcriptome profiling. Biochem Cell Biol.

[CR48] Legrand D, Elass EE, Carpentier M (2006). Interactions of lactoferrin with cells involved in immune function. Biochem Cell Biol.

[CR49] Li HY, Li M, Luo CC (2017). Lactoferrin exerts antitumor effects by inhibiting angiogenesis in a HT29 human colon tumor model. J Agric Food Chem.

[CR50] Liang J, Bi N, Wu S (2017). Etoposide and cisplatin versus paclitaxel and carboplatin with concurrent thoracic radiotherapy in unresectable stage III non-small cell lung cancer: a multicenter randomized phase III trial. Ann Oncol.

[CR51] Lönnerdal B, Iyer S (1995). Lactoferrin: molecular structure and biological function. Annu Rev Nutr.

[CR52] Lu T, Yang X, Huang Y (2019). Trends in the incidence, treatment, and survival of patients with lung cancer in the last four decades. Cancer Manag Res.

[CR53] Mantena SK, Sharma SD, Katiyar SK (2006). Berberine, a natural product, induces G1-phase cell cycle arrest and caspase-3-dependent apoptosis in human prostate carcinoma cells. Mol Cancer Ther.

[CR54] Marth JD, Grewal PK (2008). Mammalian glycosylation in immunity. Nat Rev Immunol.

[CR55] Matsumoto S, Ikura K, Ueda M (1995). Characterization of a human glycoprotein (erythropoietin) produced in cultured tobacco cells. Plant Mol Biol.

[CR56] Metz-Boutigue MH, Jollès J, Mazurier J (1984). Human lactotransferrin: amino acid sequence and structural comparisons with other transferrins. Eur J Biochem.

[CR57] Nandi S, Yalda D, Lu S (2005). Process development and economic evaluation of recombinant human lactoferrin expressed in rice grain. Transgenic Res.

[CR58] Neville MC, Zhang P (2000). Lactoferrin secretion into milk: comparison between ruminant, murine, and human milk. J Anim Sci.

[CR59] Ohtsubo K, Marth JD (2006). Glycosylation in cellular mechanisms of health and disease. Cell.

[CR60] Okubo K, Kamiya M, Urano Y (2016). Lactoferrin suppresses neutrophil extracellular traps release in inflammation. EBioMedicine.

[CR61] Parc AL, Karav S, Rouquié C (2017). Characterization of recombinant human lactoferrin N-glycans expressed in the milk of transgenic cows. PLoS One.

[CR62] Pereira CS, Guedes JP, Gonçalves M (2016). Lactoferrin selectively triggers apoptosis in highly metastatic breast cancer cells through inhibition of plasmalemmal V-H+-ATPase. Oncotarget.

[CR63] Pierce A, Colavizza D, Benaissa M (1991). Molecular cloning and sequence analysis of bovine lactotransferrin. Eur J Biochem.

[CR64] Qiao M, Jiang T, Ren S (2018). Combination strategies on the basis of immune checkpoint inhibitors in non-small-cell lung cancer: where do we stand?. Clin Lung Cancer.

[CR65] Qiu J, Zhao B, Shen Y (2013). A novel p-terphenyl derivative inducing cell-cycle arrest and apoptosis in MDA-MB-435 cells through topoisomerase inhibition. Eur J Med Chem.

[CR66] Rascón-Cruz Q, Espinoza-Sánchez EA, Siqueiros-Cendón TS (2021). Lactoferrin: a glycoprotein involved in immunomodulation, anticancer, and antimicrobial processes. Molecules.

[CR67] Redwan EM, El-Baky NA, Al-Hejin AM (2016). Significant antibacterial activity and synergistic effects of camel lactoferrin with antibiotics against methicillin-resistant *Staphylococcus aureus* (MRSA). Res Microbiol.

[CR68] Remon J, Mezquita L, Corral J (2018). Immune-related adverse events with immune checkpoint inhibitors in thoracic malignancies: focusing on non-small cell lung cancer patients. J Thorac Dis.

[CR69] Rodrigues L, Teixeira J, Schmitt F (2009). Lactoferrin and cancer disease prevention. Crit Rev Food Sci Nutr.

[CR70] Rosa L, Cutone A, Lepanto MS (2018). Physico-chemical properties influence the functions and efficacy of commercial bovine lactoferrins. Biometals.

[CR71] Safaeian L, Javanmard SH, Mollanoori Y (2015). Cytoprotective and antioxidant effects of human lactoferrin against H_2_O_2_-induced oxidative stress in human umbilical vein endothelial cells. Adv Biomed Res.

[CR72] Sawatzki G, Rich IN (1989). Lactoferrin stimulates colony stimulating factor production in vitro and in vivo. Blood Cells.

[CR73] Shental-Bechor D, Levy Y (2009). Folding of glycoproteins: toward understanding the biophysics of the glycosylation code. Curr Opin Struct Biol.

[CR74] Sorensen M, Sorensen SPL (1940). The proteins in whey. C r Trav Lab Carlsberg Ser Chim.

[CR75] Sung H, Ferlay J, Siegel RL (2021). Global Cancer Statistics 2020: GLOBOCAN estimates of incidence and mortality worldwide for 36 cancers in 185 countries. CA Cancer J Clin.

[CR76] Tung YT, Chen HL, Yen CC (2013). Bovine lactoferrin inhibits lung cancer growth through suppression of both inflammation and expression of vascular endothelial growth factor. J Dairy Sci.

[CR77] Valster A, Tran NL, Nakada M (2005). Cell migration and invasion assays. Methods.

[CR78] Wang PF, Chen Y, Song SY (2017). Immune-related adverse events associated with Anti-PD-1/PD-L1 treatment for malignancies: a meta-analysis. Front Pharmacol.

[CR79] Wang F, Wang S, Zhou Q (2020). The resistance mechanisms of lung cancer immunotherapy. Front Oncol.

[CR80] Ward PP, Piddington CS, Cunningham GA (1995). A system for production of commercial quantities of human lactoferrin: a broad spectrum natural antibiotic. Biotechnology.

[CR81] Ward PP, Cunningham GA, Conneely OM (1997). Commercial production of lactoferrin, a multifunctional iron-binding glycoprotein. Biotechnol Genet Eng Rev.

[CR82] Wormald MR, Petrescu AJ, Pao YL (2002). Conformational studies of oligosaccharides and glycopeptides: complementarity of NMR, X-ray crystallography, and molecular modelling. Chem Rev.

[CR83] Xiao Y, Monitto CL, Minhas KM (2004). Lactoferrin down-regulates G1 cyclin-dependent kinases during growth arrest of head and neck cancer cells. Clin Cancer Res.

[CR84] Yang P, Wang J, Gong G (2008). Cattle mammary bioreactor generated by a novel procedure of transgenic cloning for large-scale production of functional human lactoferrin. PLoS One.

[CR85] Yang WS, SriRamaratnam R, Welsch ME (2014). Regulation of ferroptotic cancer cell death by GPX4. Cell.

[CR86] Yoneda K, Imanishi N, Ichiki Y (2018). Immune checkpoint inhibitors (ICIs) in non-small cell lung cancer (NSCLC). J UOEH.

[CR87] Zhang Y, Nicolau A, Lima CF (2014). Bovine lactoferrin induces cell cycle arrest and inhibits mTOR signaling in breast cancer cells. Nutr Cancer.

[CR88] Zhang Y, Lima CF, Rodrigues LR (2015). In vitro evaluation of bovine lactoferrin potential as an anticancer agent. Int Dairy J.

[CR89] Zhao C, Liu Z, Fan B (2006). Differential glycosylation of rhLf expressed in the mammary gland of transgenic mice. Anim Biotechnol.

[CR90] Zimecki M, Artym J, Kocięba M (2013). Homologous lactoferrin triggers mobilization of the myelocytic lineage of bone marrow in experimental mice. Stem Cells Dev.

[CR91] Zimecki M, Actor JK, Kruzel ML (2021). The potential for lactoferrin to reduce SARS-CoV-2 induced cytokine storm. Int Immunopharmacol.

